# Time-Restricted Feeding Modifies the Fecal Lipidome and the Gut Microbiota

**DOI:** 10.3390/nu15071562

**Published:** 2023-03-23

**Authors:** Bret M. Rust, Matthew J. Picklo, Lin Yan, Aaron A. Mehus, Huawei Zeng

**Affiliations:** USDA-ARS Grand Forks Human Nutrition Research Center, Grand Forks, ND 58203, USA; brrust@iu.edu (B.M.R.); pickl014@umn.edu (M.J.P.); lin.yan@usda.gov (L.Y.); aaron.mehus@und.edu (A.A.M.)

**Keywords:** time-restricted feeding, gut microbiota, lipidomics, fecal fatty acids, bile acids, short-chain fatty acids

## Abstract

Time-restricted feeding (TRF) has been identified as an approach to reduce the risk of obesity-related metabolic diseases. We hypothesize that TRF triggers a change in nutrient (e.g., dietary fat) absorption due to shortened feeding times, which subsequently alters the fecal microbiome and lipidome. In this report, three groups of C57BL/6 mice were fed either a control diet with ad libitum feeding (16% energy from fat) (CTRL-AL), a high-fat diet (48% energy from fat) with ad libitum feeding (HF-AL), or a high-fat diet with time-restricted feeding (HF-TRF) for 12 weeks. No changes in microbiota at the phylum level were detected, but eight taxonomic families were altered by either feeding timing or dietary fat content. The HF-AL diet doubled the total fecal fatty acid content of the CTRL-AL diet, while the HF-TRF doubled the total fecal fatty acid content of the HF-AL diet. Primary fecal bile acids were unaffected by diet. Total short-chain fatty acids were reduced by HF-AL, but this effect was diminished by HF-TRF. Each diet produced distinct relationships between the relative abundance of taxa and fecal lipids. The anti-obesogenic effects of TRF in HF diets are partly due to the increase in fat excretion in the feces. Furthermore, fat content and feeding timing differentially affect the fecal microbiota and the relationship between the microbiota and fecal lipids.

## 1. Introduction

Obesity is a serious chronic condition associated with several comorbidities (cardiovascular disease, non-alcoholic fatty liver disease, diabetes), and health costs associated with these diseases were estimated to be $173 billion in 2019 [1]. An effective approach to reducing adiposity and its associated negative health consequences is to limit food availability to no more than 10 to 12 h per day, known as time-restricted feeding or eating (TRF or TRE) [2,3,4,5].

The gut microbiome is altered by TRF [6,7]. Fecal microbial taxonomy oscillates diurnally and is controlled by the host circadian clock [8,9]. In these studies, TRF governed daily oscillation in microbiota composition while improving metabolic health. TRF promotes metabolic homeostasis by regulating macronutrient availability and the circadian clock [10,11]. Moreover, dietary fat alters the gut microbiota [7,12,13,14,15,16,17,18]. However, the mechanisms underlying the relationship between dietary fat intake, TRF, and the fecal lipidomic profile remain unexplored. Therefore, the link between improved metabolic health from TRF may lie in further understanding fecal lipidomics.

Saturated fatty acid intake is associated with obesity, and an average American diet contains 11% of energy derived from saturated fatty acids [19,20]. Saturated fatty acids have an anti-microbial effect that acts on the cell membranes of bacteria [21,22]. Recent reports have suggested that fat content rather than obesity itself alters the gut microbiota [23]. However, other data have shown obesity to stimulate the production of anti-microbial peptides from the host [24]. Understanding the interaction between dietary fat, obesity, and TRF will shed light on the anti-obesigenic effects and improvements in glucose homeostasis and hepatic steatosis we previously demonstrated in TRF [25] and if those benefits are mediated by the gut microbiota or lipid metabolites produced from the gut microbiota.

The gut microbiota produces a variety of lipid metabolites that affect host metabolism [26,27]. Bile acids and short-chain fatty acids have been studied extensively regarding the gut microbiota and, to some extent, in their response to TRF. Because the antimicrobial effects of fatty acids are specific to taxa, and the taxonomy of the gut microbiota is tied to glucose homeostasis and obesity, the makeup and the content of fecal fatty acids have implications for metabolic health [26,27]. However, fecal fatty acid content and how it relates to the gut microbiota and other fecal lipids have been studied little. In our previous work, insulin sensitivity and body weight were improved by TRF, although there was little difference in food intake between the HF-AL and HF-TRF groups [25]; we hypothesize that TRF triggers a change in nutrient (e.g., dietary fat) absorption due to shortened feeding times, which may explain the improvements in metabolic health because of TRF and will lay the foundation for future studies examining host metabolic health.

In this project, we evaluated the effect of ad libitum HF feeding and TRF on (1) the fecal microbiota determined by 16S rRNA sequencing, (2) fecal lipid content, and (3) the relationship between the fecal microbiota and the fecal lipidome consisting of fatty acids, bile acids, and short-chain fatty acids. These data on the fecal lipidome will provide a mechanistic backdrop linked to the fecal microbiota.

## 2. Materials and Methods

### 2.1. Materials

Bile acid standards were ordered from Steraloids Inc. (Newport, RI, USA). Nonadecanoic acid, methyl nonadecanoate, and fatty acids mixtures GLC 462 and GLC 569 were ordered from Nuchek Prep. (Elysian, MN, USA). BCFA (14-methylhexadecanoic acid (14-Me16:0), 12-methyltridecanoic acid (12-Me13:0), 16-methylheptadecanoic acid (16-Me17:0), 18-methyleicosanoic acid (18-Me20:0), 14-methylpentadecanoic acid (14-Me15:0), 12-methyltetradecanoic acid (12-Me14:0), 15-methylhexadecanoic acid (15-Me16:0), and 13-methyltetradecanoic acid (13-Me14:0)) were ordered from Matreya LLC, (State College, PA, USA) and Larodan AB, (Solna, Sweden). In some cases, peak identity was inferred by retention time due to the lack of a purified standard.

### 2.2. Animal Experimentation

The study design has been described and was approved by the Institutional Animal Care and Use Committee of the USDA-ARS Grand Forks Human Nutrition Research Center [25]. Briefly, 42 male, C57BL/6 mice aged 12 weeks were fed AIN93 G-based diets for 12 weeks. Animals were divided into three groups (14/group). Mice were fed either an ad libitum (AL) control diet (CTRL) providing 16% of energy from fat, an AL high-fat diet (HF) (48% energy fat), or a time-restricted feeding (TRF) HF diet. TRF was limited to the dark phase between Zeitgeber times (ZT) 12 and 24.

Animals were acclimated in a pathogen-free room (light-dark cycle: 12/12 h, room temperature: 22 ± 1 °C) for 4 weeks, then divided equally by weight into 3 groups, housed individually, and for 12 weeks fed AIN93G-based diets similar to those used in previous studies of obesity [20,28]. The energy content of the CTRL diet (4.1 kcal/g) was 16% energy as fat, 20% energy protein, and 64% energy as carbohydrate, while the energy content of the HF diets (4.9 kcal/g) was 48% energy fat, 20% energy protein, and 30% energy carbohydrate. The energy content ratio of linoleic acid (LA; 18:2*n*-6) to α-linolenic (ALA; 18:3*n*-3) is 5:1 in each diet.

Fecal pellet collection was performed 3–4 consecutive days beginning one week prior to the end of the study. At ZT, 12 fresh cardboard trays were placed under each animal’s cage. At ZT 3 the following day, cardboard was gently removed, and pellets were collected using clean forceps into 2 mL Eppendorf tubes for each animal. Pellets on the boundary of adjacent cages were excluded. After collection, pellets were immediately frozen at −80 °C until analyzed.

### 2.3. Fecal Lipid Analysis

Fecal fatty acid composition was determined by transesterification of extracted fecal lipids in acidified methanol. Fatty acid methyl ester (FAME) derivatization was performed directly on pulverized freeze-dried fecal sample (approximately 50 mg) by the addition of FAME reagent (2 mL, anhydrous methanol: acetyl chloride, 19:1, *v*/*v*) and 100 µL of 14 mM nonadecanoic acid in anhydrous methanol as an internal standard [20,29]. Samples were incubated at 35 °C overnight on a rotator. The reaction was quenched with 500 µL of 1.4 M potassium carbonate solution. Fatty acid methyl esters were extracted in 1 mL hexane.

Fatty acid methyl esters were analyzed by GC-FID on a Thermo 1310 Trace gas chromatograph (Thermo Scientific, Waltham, MA, USA) using an SP-2560 capillary GC Column (75 m × 0.18 mm, 0.14 µm (Millipore Sigma, Burlington, MA, USA). The injector was operated with a 50:1 split with a 2 mL flow of hydrogen carrier gas on column. The injector and detector were held at 250 °C and 270 °C, respectively. The temperature profile began at 50 °C, held for 1 min, then was raised at 5 °C/min to a final temperature of 240 °C where it was held for 1 min. In each batch of samples, fatty acids species were identified by retention time alignment using quality control standards generated from two commercially available FAMES mixtures, GLC 462 and GLC 569 (Nuchek Prep, Elysian, MN, USA), and an in-house reference solution containing branched-chain fatty acids. Response factors were determined using GLC 462 in hexane at three concentrations (0.93, 9.29, and 92.9 mg/mL in each fatty acid component) and one level of GLC 569 (7.35 mg/mL in each fatty acid component). In cases where only one concentration was available, for example, odd-chain and branched-chain fatty acids, the response factor of a near-neighbor with the same number of carbons was assigned. The response of the assigned species was used to calculate the analyte concentration. The calculated concentration value was compared to the stated concentration for quality control purposes. Calibration sets were run concurrently with each sample batch.

Short-chain fatty acid (SCFA) analysis: analysis of fecal SCFAs has been described [30]. Briefly, freeze-dried feces were pulverized using a TissueLyser (Qiagen, Valencia, CA, USA), and ~25 mg was suspended in 1 mL water with 100 µL of ethyl butyric acid internal standard solution (5 mmol/L). The suspension was acidified with 65 µL of 6 molar hydrochloric acids combined with 1 mL of methyl tert–butyl ether. Samples were mixed using a vortex mixture and then centrifuged at 17,000× *g* for 20 min at 4 °C. The ether layer was collected and analyzed for SCFA using a Thermo Trace-1310 gas chromatograph with flame ionization detection (Thermo Fisher, Waltham, MA, USA) and DB-FFAP column (30 m × 0.530 mm × 0.5 µm; Agilent Technologies Inc., Santa Clara, CA, USA). SCFA species were quantitated by peak area relative to the internal standard and normalized to the mass fecal material used.

### 2.4. Fecal Bile Acid Analysis

The analysis of fecal bile acids (BA) has been described [30]. In brief, feces were collected during the last week of the feeding period and stored at −80 °C. Samples were pulverized as described above, and ~25 mg of material was suspended in 1 mL 1:1 ethanol: acetonitrile solution along with 100 µL of internal standard solution (23-nordeoxycholic acid, 175 ng/tube). Samples were mixed into a slurry using a TissueLyser (Qiagen, Valencia, CA, USA) with shaking or disruption for 10 min, then incubated at 60 °C for one hour before centrifugation (4 °C, 13,000 g, 5 min). The supernatant was collected, and the pellet was re-extracted with an additional 1 mL of 1:1 ethanol: acetonitrile. After centrifugation, the second supernatant was combined with the first, and solvent was removed under gentle stream of argon in a 30 °C water bath. The sample was reconstituted in 100 µL of 9:1 water: methanol (20 mM solution of ammonium acetate). The BA composition was measured by mass spectrometry coupled to liquid chromatography with electrospray ionization as previously described [31].

### 2.5. Fecal Microbiome Analysis

The University of Minnesota Genome Center (UMGC, Minneapolis, MN, USA) performed extraction and sequencing of 16S rRNA. RNA was extracted from mouse fecal pellets (50 mg) using a bead-beating method and a MoBio DNA extraction kit. The V3-V4 regions of 16S rRNA were sequenced on an Illumina (San Diego, CA, USA) MiSeq stowaway sequencing run. UMGC used a dual indexing sequencing approach of V3V4 regions with KAPA HiFi polymerase that has been previously described [32]. Raw FASTQ data are demultiplexed and trimmed using QIIME 2 software and then denoised with Dada2 software. These data were uploaded into MicrobiomeAnalyst and were filtered so that at least 20% of values would contain at least 4 features. Results were similar when data were rarefied and not rarefied; due to the low variability of the sequencing depth, we used the unrarefied data in accordance with the recommendations of the software. Alpha and beta diversity were determined from feature-level analysis [33,34].

### 2.6. Statistical Analysis

Statistical analyses were performed on JMP 15.0.0 statistical software (SAS Institute, Cary, NC, USA). Relative abundances of taxa to the level of family were calculated from OTU read counts at each taxonomic level and exported from MicrobiomeAnalyst for analysis in JMP 16.1.0 statistical software (SAS Institute, Cary, NC, USA) [33,34]. We used two measures of alpha diversity to encompass the effect of major taxonomic classifications using the Shannon Index and less abundant taxonomy with the Chao1 Index because TRF may alter the relative abundances of mucosal layer taxa that are less abundant [35]. Ranks averages were determined for the relative abundance of each taxonomic level (% of total bacterial abundance) and for the fecal concentration of each lipid class. Spearman’s Rho correlation coefficients were calculated from rank averages between each lipid class and each taxonomic level to family. One-way ANOVA was performed to determine the effects of diet on normally distributed data. We used Tukey’s post hoc analysis to determine specific differences between diets and contrast tests to determine the effect of fat level or feeding schedule. Nonparametric Steel–Dwass was performed on data that were not normally distributed. *p*-values less than 0.05 were considered statistically significant.

## 3. Results

### 3.1. Fecal Bacterial Taxa

**Figure 1:**
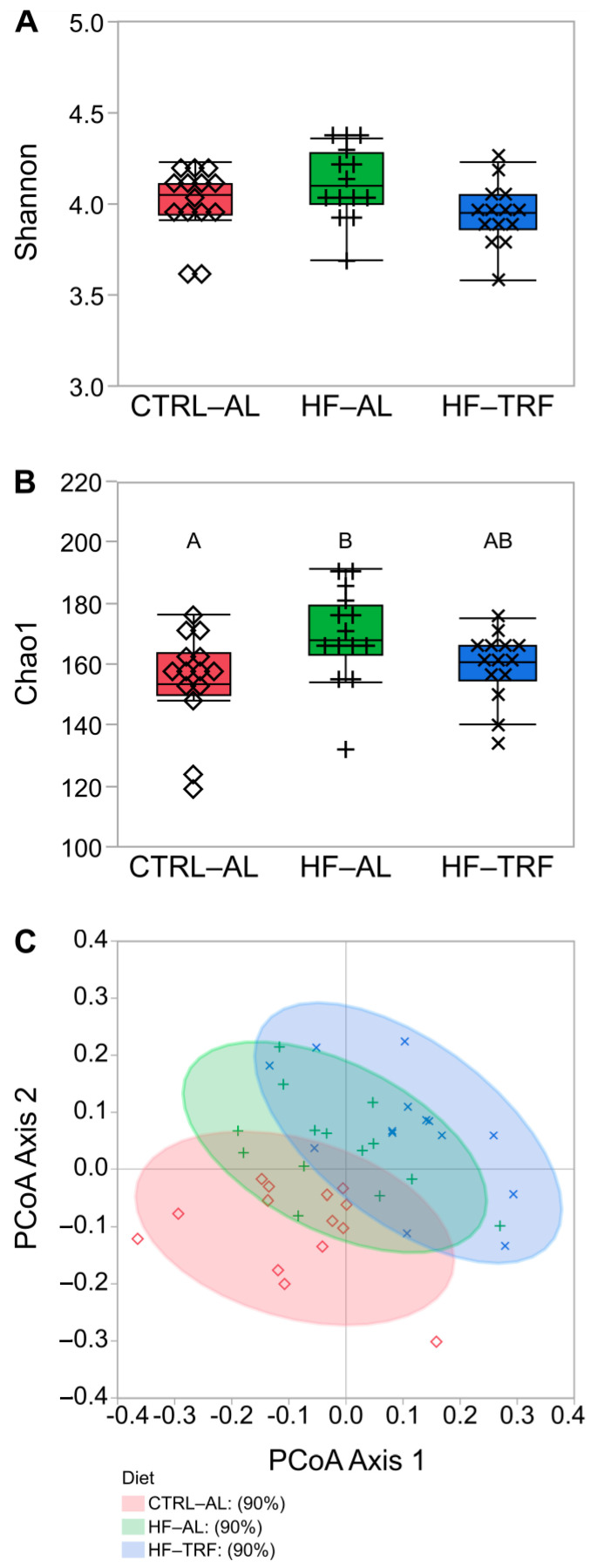
Alpha diversity indices and beta diversity using principal coordinates analysis. Like superscripts (A, or B) are not statistically different. (N = 14 for each diet). (**A**) Fecal microbiota alpha diversity did not differ between diets in the Shannon Diversity Index following 12 weeks of either CTRL-AL, HF-AL, or HF-TRF diets in C57BL/6 mice. (**B**) Fecal microbiota alpha diversity was increased by HF-AL feeding compared to CTRL-AL feeding in the Chao1 Index (*p* = 0.02), but HF-TRF did not differ from either AL diet. One-way ANOVA with Tukey’s post hoc test for each analysis. (**C**) Beta diversity principal coordinates plot fecal microbiota beta diversity. Beta diversity shows HF diets adjacent to each other and AL diets adjacent to each other, while the greatest separation exists between CTRL-AL and HF-TRF. CTRL-AL, control ad libitum feeding; HF-AL, high-fat ad libitum feeding; HF-TRF, high-fat time-restricted feeding; PCoA, principal coordinate analysis.

Feature-level alpha diversity did not differ using the Shannon Index (Figure 1A). However, as measured by Chao1, HF increased the microbial richness and the number of the different types of organisms in an ecosystem, but TRF abolished this effect (Figure 1B). Analysis of beta diversity, comparing the microbial community between treatments, demonstrated clustering by treatment type (Figure 1C). Samples clustered by diet with the AL groups and HF groups adjacent. CTRL-AL clustered mostly in the lower left quadrant, while HF-TRF clustered mostly in the upper and right halves of the figure.

No differences were found between treatments in the relative abundance of Firmicutes and Bacteroidetes. Nonparametric tests demonstrated an elevated relative abundance of Cyanobacteria in the CTRL-AL diet compared to both HF diets and an elevated relative abundance of Patescibacteria in CTRL-AL compared to HF-TRF. There was a strong inverse relationship between the relative abundances of Firmicutes and Bacteroidetes, but this relationship was unaffected by diet, and the ratio of these two most abundant phyla was unchanged by diet.

**Figure 2:**
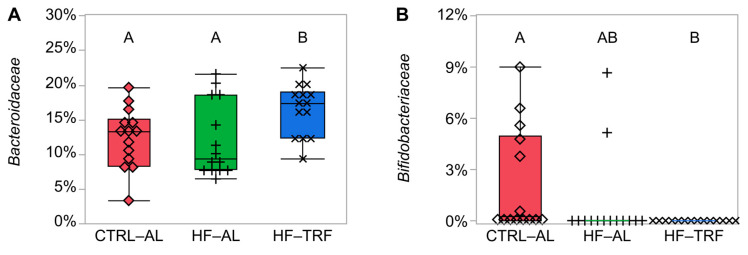

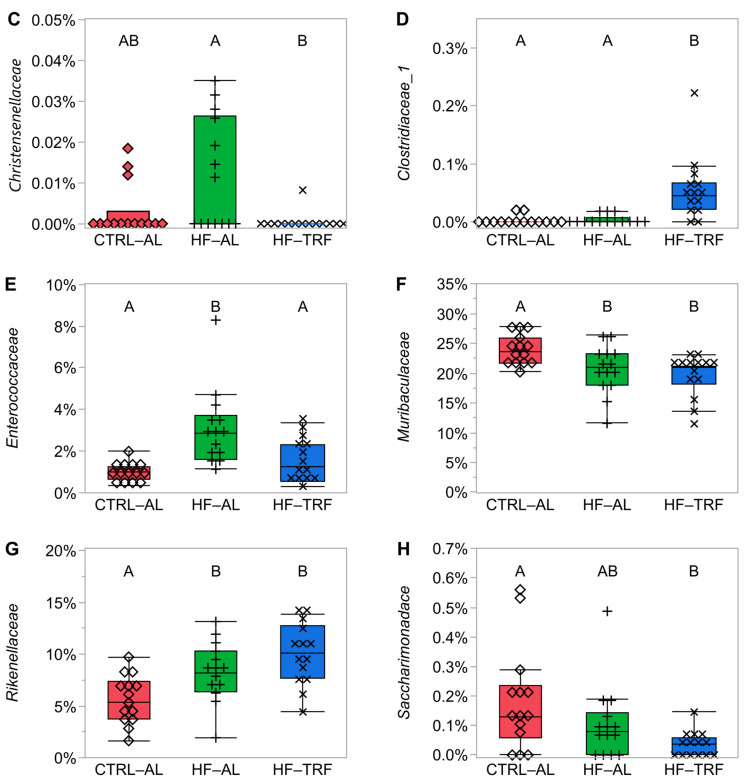
Relative abundance of families by diet. Like superscripts (A, and B) are not statistically different. 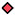 each animal within LF-AL; 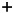 indicates individual animals within HF-AL; 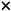 indicates each animal in HF-TRF (N = 14 for each diet). (**A**) * Bacteroidaceae were elevated in HF-TRF compared to CTRL-AL and HF-AL diets (*p* = 0.02). (**B**) ** Bifidobacteriaceae were not detected in mice undergoing HF-TRF feeding, and relative abundance was lower than CTRL-AL (*p* < 0.01). Two animals in the HF-AL had detectable OTUs attributable to Bifidobacteriaceae, but no differences were seen between the HF-AL and CTRL-AL or HF-TRF animals. (**C**) ** Christensenellaneae were elevated by HF-AL feeding compared to HF-TRF (*p* = 0.03), but animals with CTRL-AL feeding did not differ from HF-AL or HF-TRF. (**D**) ** Clostridiaceae were increased by HF-TRF compared to both CTRL-AL and HF-AL diets (*p* < 0.01 for both). (**E**) ** Enterococcaceae were increased by HF-AL feeding compared to CTRL-AL and HF-TRF (*p* < 0.01 and *p* = 0.04, respectively). (**F**) * Muribaculaceae were increased by CTRL-AL feeding compared to HF-AL and HF-TRF (*p* = 0.03 and *p* < 0.01, respectively). (**G**) * Rikenellaceae were higher in HF-AL and HF-TRF than in CTRL-AL (*p* = 0.04 and *p* < 0.01, respectively). (**H**) Saccharimonadaceae was reduced by HF-TRF compared to CTRL-AL (*p* = 0.02), but HF-AL did not differ from either CTRL-AL or HF-TRF. * One-way ANOVA with Tukey’s post hoc test. ** Steel–Dwass nonparametric test. CTRL-AL, control ad libitum feeding; HF-AL, high-fat ad libitum feeding; HF-TRF, high-fat time-restricted feeding. The asterisks are intended to denote which statistical tests were applied to each taxonomic family: non-parametric for non-Gaussian residual error and parametric for families with normally distributed residual error.

We compared relative abundance only to the level of the family because the inability of 16S rRNA, even in the V3-V4 region, to resolve taxa diminishes at lower taxonomic levels [36,37]. Several taxonomic families were altered by diet (Figure 2A–H). HF-TRF elevated the relative abundance of *Bacteroidaceae* over both HF-AL and CTRL-AL. *Bifidobacteriaceae* were not detected in HF-TRF, and nonparametric tests showed the relative abundance in CTRL-AL to be elevated with respect to HF-TRF but did not reach significance with respect to HF-AL (*p* = 0.06). *Christensenellaceae*, although of low relative abundance overall, were detected in only one mouse in HF-TRF, three mice in the CTRL-AL group, but in seven mice in HF-AL with nonparametric tests showing HF-TRF relative abundance lower than HF-AL. In contrast, *Clostridiaceae* 1 were elevated in HF-TRF compared to both AL groups. *Enteroccoccaceae* were elevated in HF-AL relative to CTRL-AL and HF-TRF. HF diets lowered *Muribaculaceae* compared to CTRL-AL, while HF diets raised *Rikenellaceae* compared to CTRL-AL. The CTRL-AL diet raised *Sacharimonadace* compared to HF-TRF but not HF-AL.

### 3.2. Fecal Lipid Content

#### 3.2.1. Fecal Fatty Acids

**Figure 3:**
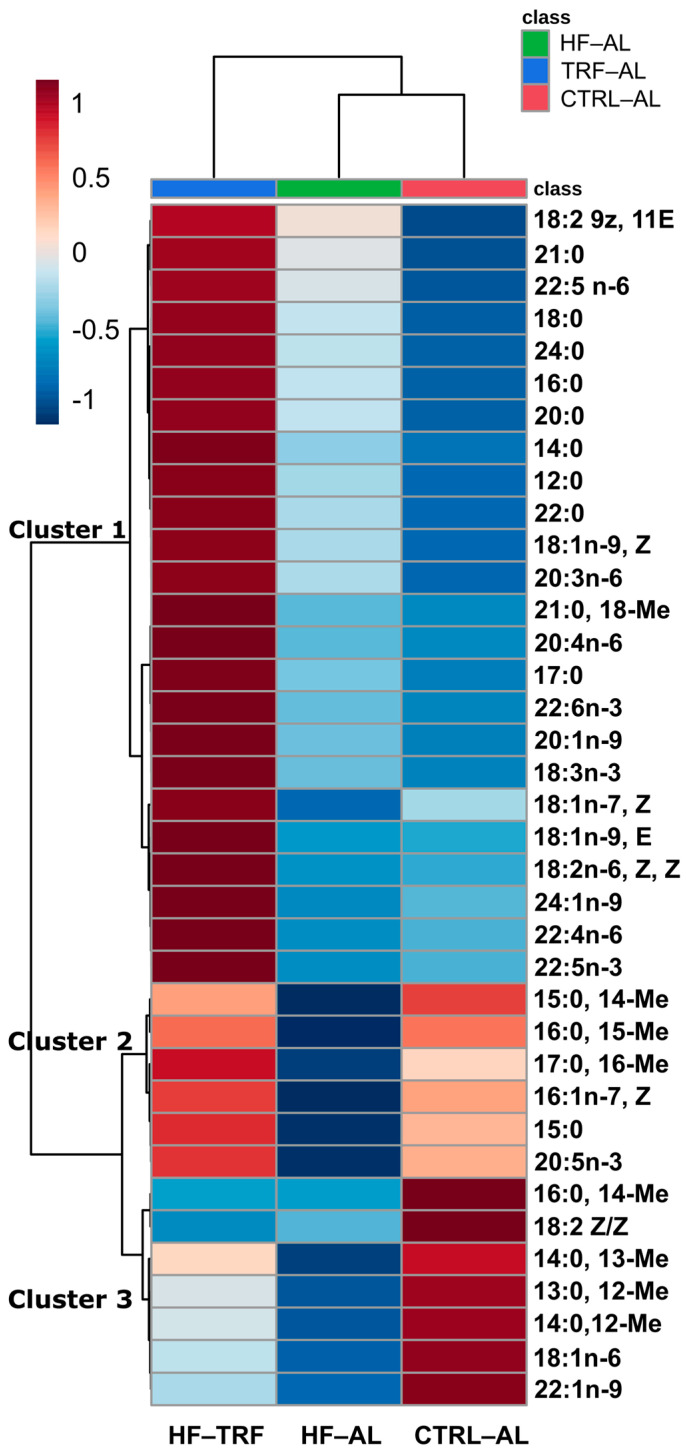
Heatmap and hierarchical clustering of fecal fatty acids. Cluster 1 highlights the increased levels of SFA, MUFA, and some PUFA species in HF-TRF compared to both the HF-AL and CTRL-AL diets, while Cluster 2 features diminished BCFA species in the HF-AL diet compared to both CTRL-AL and HF-TRF. Cluster 3 is characterized by elevated species of BCFA in the CTRL-AL diet and decreased relative to HF-AL. BCFA, branched-chain fatty acids; CTRL-AL, control ad libitum; HF-AL, high-fat ad libitum; HF-TRF, high-fat time-restricted feeding. MUFA, monounsaturated fatty acids; PUFA, polyunsaturated fatty acids; SFA, saturated fatty acids.

Hierarchical clustering with heat mapping (Figure 3) was used to identify treatment-dependent changes in the fecal content of fatty acids. Three main clusters were identified that include fatty acids that were, in general, (1) HF-TRF> HF-AL > CTRL-AL, which represented the majority of fatty acids, (2) HF-TRF > CTRL-AL > HF-AL, and (3) CTRL-AL > HF-TRF > HF-AL. Fatty acids in cluster 2 and cluster 3 were predominantly branched-chain (iso and ante-iso) fatty acids (BCFA).

**Figure 4:**
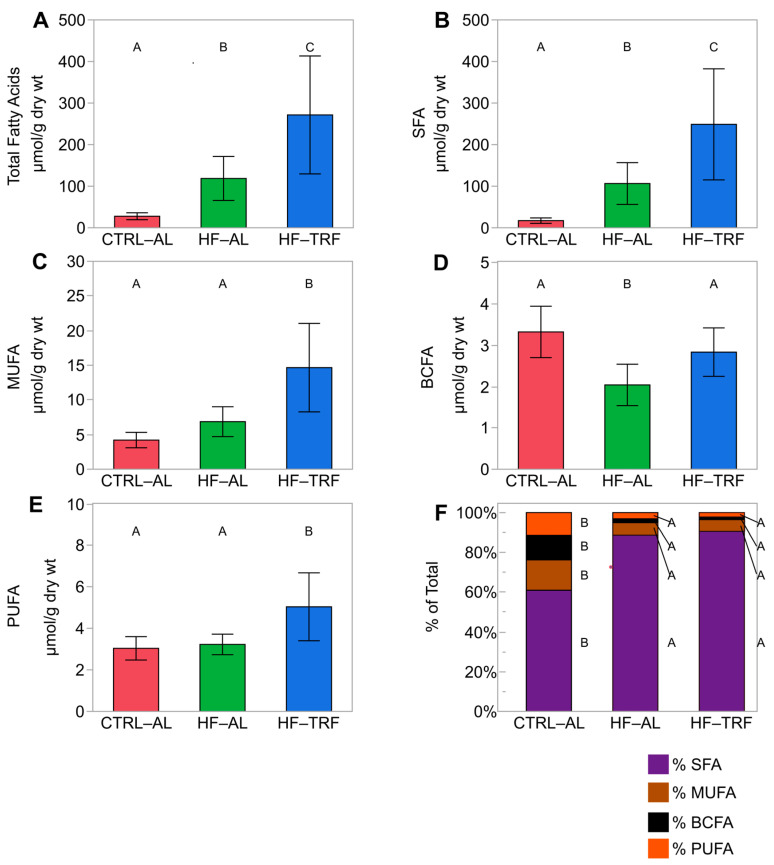
Fecal fatty acid types by diet. Like superscripts (A, B, and C) are not statistically different within each chemical type. (N = 14 for each diet) (mean ± SD). (**A**) Total fecal fatty acids were doubled by HF-AL over CTRL-AL (*p* = 0.02), and HF-TRF doubled the total fatty acid content of HF-AL (*p* < 0.01). (**B**) Fecal SFA content mirrored the pattern seen with total fatty acids with CTRL-AL < HF-AL < HF-TRF (*p* < 0.01 for each). (**C**) Fecal MUFA were elevated in HF-TRF compared to both CTRL-AL and HF-AL (*p* < 0.01 for both). (**D**) Long-chain BCFA (>C12) were elevated in the feces of CTRL-AL and HF-TRF over HF-AL (<0.01 for each). (**E**) Fecal PUFA were also elevated in HF-TRF compared to both CTRL-AL and HF-AL (*p* < 0.01 for both). (**F**) The percent abundances for each type of fecal fatty acid by diet. % SFA was nearly identical in both HF diets and was greater than CTRL-AL (*p* < 0.01 for each). Fecal MUFA, long-chain BCFA, and PUFA% abundance were elevated in CTRL-AL compared to both HF diets (*p* < 0.01 for all). One-way ANOVA with Tukey’s post hoc test for each analysis. CTRL-AL, control ad libitum; HF-AL, high-fat ad libitum; HF-TRF, high-fat time-restricted feeding; BCFA, branched-chain fatty acids; MUFA, monounsaturated fatty acids; PUFA, polyunsaturated fatty acids; SFA, saturated fatty acids.

Total fecal fatty acid content differed among all three diets such that HF-AL elevated fecal fatty acids vs. the CTRL-AL (120 ± 53 µmol/g vs. 29 ± 8 µmol/g) and TRF increased total fecal fatty acids (273 ± 142 µmol/g) vs. HF-AL (Figure 4A). For the purpose of this analysis, we differentiate between BCFA and SFA and between BCFA and branched short-chain fatty acids. HF-AL increased the fecal content of total SFA, and this effect was enhanced by TRF (Figure 4B). MUFA were elevated in HF-TRF compared to both AL diets (Figure 4C). BCFA represented 1.7 ± 0.9% of the fecal fatty acids produced in the HF diets and about 12.4 ± 2.0% in the CTRL-AL diet. Total BCFA are reduced by HF-AL but are restored by TRF (Figure 4D). The content of total polyunsaturated fatty acids was increased by TRF over AL and did not differ between AL diets (Figure 4E). Notably, the percentage of all fecal fatty acid types was nearly identical in both HF diets. SFA were 88.7 ± 2.7% for HF-AL and 90.6 ± 3.0% for HF-TRF, in which the percent SFA was elevated relative to CTRL-AL, 61.0 ± 4.4%, while the percentage of MUFA, BCFA, and PUFA were all elevated in CTRL-AL compared to both HF diets (Figure 4F).

#### 3.2.2. Fecal Bile Acids

**Figure 5:**
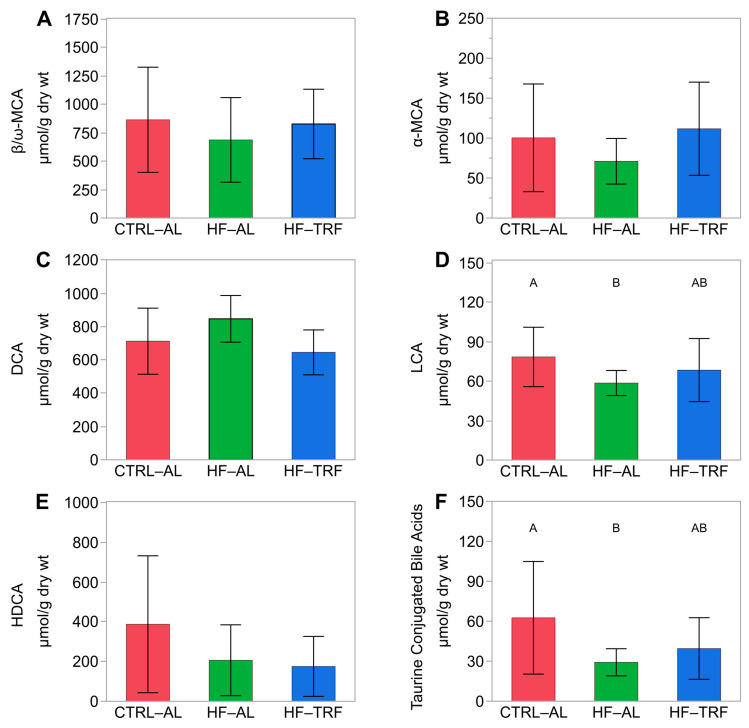
Fecal bile acids by diet. Like superscripts (A and B) are not statistically different. (N = 14 for each diet) (mean ± SD). (**A**) Combined β and ω MCA did not differ between diets. (**B**) α-MCA did not differ between diets. (**C**) DCA did not differ between diets. (**D**) LCA was elevated in the CTRL-AL diet compared to HF-AL (*p* = 0.03). HF-TRF did not differ from either AL diet. (**E**) HDCA did not differ with diet. (**F**) Taurine conjugates were elevated in CTRL-AL compared to HF-AL. Taurine conjugates in HF-TRF did not differ from either AL diet. One-way ANOVA with Tukey’s post hoc test for each analysis. CTRL-AL, control ad libitum; HF-AL, high-fat ad libitum; HF-TRF, high-fat time-restricted feeding. MCA, muricholic acid; HDCA, hyodeoxycholic acid; DCA, deoxycholic acid; LCA, lithocholic acid.

The most abundant fecal bile acids after 12 weeks of feeding were β/ω MCA and DCA (827.3 ± 305.8 μM and 644.6 ± 134.7 μM, respectively). Total fecal bile acids, β/ω-MCA (Figure 5A), and α-MCA (Figure 5B) concentrations were not different between diets. However, DCA did not differ between CTRL-AL and HF-AL, and TRF (Figure 5C). HF-AL reduced LCA compared to CTRL-AL, but this difference was not evident in TRF (Figure 5D). HDCA did not differ between diets (Figure 5E). Notably, HF-AL decreased fecal concentrations of total taurine conjugates, but these effects were not apparent in HF-TRF.

#### 3.2.3. Fecal Short Chain Fatty Acids

**Figure 6:**
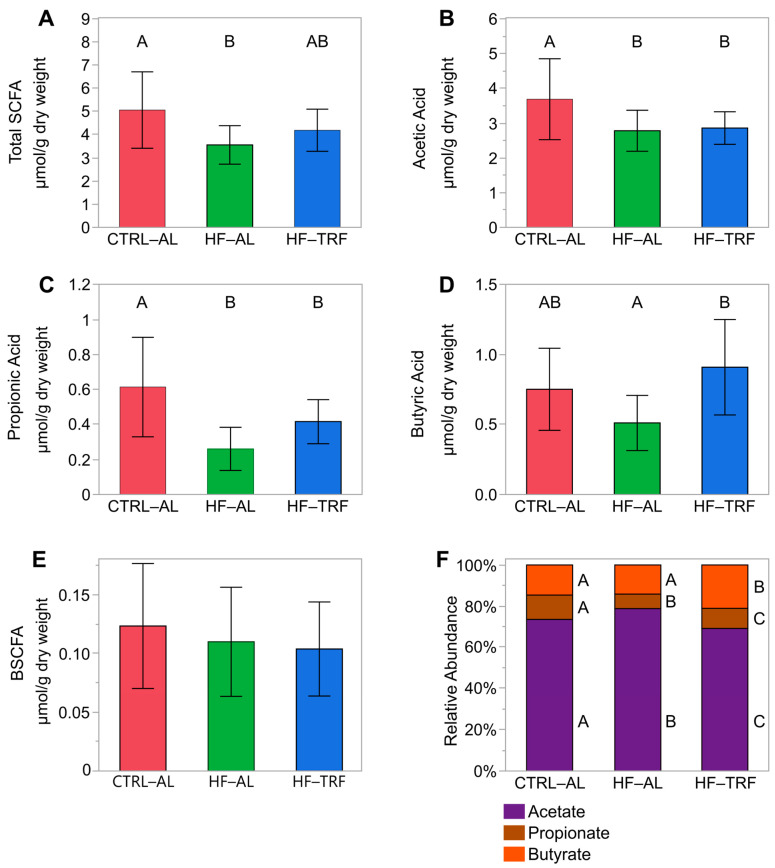
Fecal short-chain fatty acids. Like superscripts (A, B, and C) are not statistically different. (mean ± SD) (N = 14 for each diet). (**A**) Total SCFAs were greater in CTRL-AL compared to HF-AL (*p* < 0.01) but did not differ in HF-TRF from either AL diet. (**B**) Acetate was elevated in the feces of CTRL-AL compared to HF-AL and HF-TRF (*p* = 0.01 and *p* = 0.02, respectively). (**C**) Fecal propionate was greater in CTRL-AL compared to HF-AL and HF-TRF (*p* < 0.01 and *p* = 0.03, respectively). (**D**) Butyrate in the feces of HF-TRF was elevated compared to HF-AL (*p* < 0.01). Butyrate in CTRL-AL did not differ from either HF diet. (**E**) Branched-chain short-chain fatty acids did not differ between diets. One-way ANOVA with Tukey’s post hoc test for each analysis. CTRL-AL, control ad libitum; HF-AL, high-fat ad libitum; HF-TRF, high-fat time-restricted feeding. (**F**) The relative abundance of acetate was greatest in HF-AL and differed from both CTRL-AL (*p* < 0.01) and HF-TRF (*p* < 0.01). Percent acetate in CTRL-AL feces was also higher than HF-TRF (*p* = 0.03). Conversely, the percentage of fecal propionate was lowest in HF-AL and differed from both the CTRL-AL (*p* < 0.01) and HF-TRF (*p* < 0.01). CTRL-AL fecal propionate was greater than HF-TRF (*p* = 0.05). The percent butyrate was elevated in HF-TRF compared to both AL diets (*p* < 0.01 for both). CTRL-AL, control ad libitum; HF-AL, high-fat ad libitum; HF-TRF, high-fat time-restricted feeding; SCFA, short-chain fatty acids; BSCFA, branched short-chain fatty acids.

The total of the primary fermentation products, acetate, propionate, and butyrate (Figure 6A), was elevated by CTRL-AL relative to HF-AL but did not differ in HF-TRF. Fecal concentrations of acetate and propionate were elevated by CTRL-AL compared to both HF diets (Figure 6B,C). Fecal butyrate was elevated by TRF compared to HF-AL but not compared to CTRL-AL (Figure 6D). The sum of branched short-chain fatty acids, methyl valerate, and methyl butyrate did not differ between diets (Figure 6E). The relative abundance of acetate differed between each diet: HF-AL > CTRL-AL > HF-TRF. The relative abundance of propionate also differed in each diet: CTRL-AL > HF-TRF > HF-AL. The relative abundance of butyrate was elevated in HF-TRF over both CTRL-AL and HF-AL.

### 3.3. Relationship of Fecal Lipids to Fecal Microbes

#### 3.3.1. Fatty Acids

Spearman rank correlations between fecal fatty acids and phyla are presented in Supplementary Table S1, while relationships between fatty acids and taxonomic families are presented in Supplementary Table S2. At the phylum level, Actinobacteria were negatively associated with total SFA, MUFA, *n*-6 PUFA, and total fatty acids in the HF-AL diet only. This relationship is driven by *Bifidobacteriaceae*. Ad libitum feeding produced associations between the total of most types of fatty acids and the relative abundance of Bacteroidetes. This effect was driven primarily by the family *Bacteroidaceae*, but BCFA were also associated with the family *Tannerellaceae* in the ad libitum diets. Deferribacteres were inversely related to total SFA, BCFA, MUFA, and total fatty acids in the CTRL-AL diet and sporadically in HF-TRF. Ad libitum feeding showed inverse relationships between Firmicutes and BCFA, while Verrucomicrobia was positively associated with BCFA in the HF diets. Additionally, within the phylum Firmicutes, a strong positive correlation was found only in the HF-TRF diet between the taxonomic family *Clostridiaceae* and SFA, MUFA, and both types of PUFA but not with BCFA. Although there was an inverse relationship between Firmicutes and BCFA in the AL diets and a trend in HF-TRF (*p* = 0.049, *p* = 0.049, and *p* = 0.08, respectively) at the level of the family, this relationship was apparent only in *Lachnospiraceae* in the CTRL-AL diet. The sulfate-reducing *Desulfovibrionaceae* was inversely correlated with BCFA in CTRL-AL.

#### 3.3.2. Bile Acids

Spearman’s rho coefficients between bile acids and relative abundances of phyla and families to are presented in Supplementary Tables S3 and S4, respectively. The phylum Firmicutes was positively associated with many fecal bile acids, particularly CA and its conjugates in the HF-TRF group. Within Firmicutes, the taxonomic family *Clostridiales* family XIII correlated with DCA, t-HDCA, t-CDCA, t-CA, and total bile acids in the HF-TRF group. Notably, taurine conjugates were positively associated with *Lachnospiraceae* in HF-TRF. Bacteroidetes were inversely correlated with total taurine conjugates in the HF diets. These relationships were apparent at the family level in *Bacteroidaceae* and in *Tannerellaceae* with the HF-TRF diet. Importantly, the most abundant fecal bile acids were not strongly correlated to microbial phyla. However, the relative abundance of Patescibacteria was inversely correlated to each conjugate of MCA in CTRL-AL. DCA, α-MCA, and total bile acids were inversely correlated to Verrucomicrobia in HF-TRF. Actinobacteria in the HF-AL diet were strongly correlated with CA and most of its conjugate forms.

#### 3.3.3. Short-Chain Fatty Acids

Relationships between SCFA and phyla and families are depicted in Supplementary Tables S5 and S6, respectively. Phylum level relative abundance in the fecal microbiota demonstrated relationships primarily in the CTRL-AL group in which Bacteroidetes were positively associated with concentrations of all SCFAs except acetate resulting in a negative relationship with the relative abundance of acetate in Bacteroidetes. Conversely, Firmicutes produced an inverse relationship with concentrations of all SCFAs except isovalerate in only the CTRL-AL diet. However, no single family within Firmicutes could be attributed to the inverse relationship between this phylum and SCFA concentrations. A few notable relationships between families and fecal SCFAs appeared. *Bacteroidaceae* was associated with SCFAs in the CTRL-AL diet. In the HF-AL, *Eggerthellaceae*, *Flavobacteriaceae*, and *Clostridiales* Family XIII were inversely correlated to total SCFAs. *Desulfovibrionaceae* was inversely correlated to propionate, butyrate, and the branched short-chain fatty acids driving an inverse relationship to total SCFAs within the CTRL-AL diet only. The relative abundances of acetate and butyrate were positively and negatively associated, respectively, with the abundance of *Desulfovibrionaceae*. *Tannerellaceae* was associated with all SCFAs in the CTRL-AL diet.

## 4. Discussion

The current work is a follow-up to our previous findings that TRF mitigates body weight gain and detriments to glucose homeostasis from HF-AL [25]. We found that TRF reduces the absorption of dietary fat (particularly a high saturated fat diet), which subsequently changes the fecal microbiome and lipidome. Our findings (1) provide new mechanistic insights into the TRF-driven improvement in obesity-related metabolic diseases; (2) unveil a complex network of relationships between taxonomy and fecal lipids that is influenced by dietary fat content and feed timing.

### 4.1. Diet and TRF Altered the Composition of Fecal Microbiota

High-fat feeding increased the gut bacterial α-diversity according to the Chao1 index but not the Shannon α-diversity index, which is in agreement with the previous report [38]. However, TRF reduced the effects of the HF diet on the Chao1 index, which places greater weight on less abundant species [35]. This result could reflect a reduction in taxa not adapted to oscillations in energy availability and an increase in the relative abundance of taxa dependent on energy from the mucosal layer. In a principal coordinates plot of beta diversity, there was a greater separation between HF-TRF and CTRL-AL than between the CTRL-AL diet and the HF-AL diet, indicating that TRF creates a distinct taxonomic profile of the fecal microbiota from AL.

The relative abundance and the ratio between Firmicutes and Bacteroidetes were unaffected by feeding timing or fat content, which agrees with studies that have failed to show that obesogenic HF diets affect the relative abundance of phyla [39,40]. At the family level, we observed differences between HF-TRF and HF-AL in eight taxa overall, four of which differed between HF-AL and HF-TRF. In particular, *Christensenellaceae* (Firmicutes), which is associated with positive health outcomes and reduced BMI in humans [41] and was reduced in TRF from HF-AL, and *Bacteroidaceae* (Bacteroidetes) was elevated by HF-TRF compared to the AL diets. These data indicate that, although TRF does moderate the obesigenic effects of an HF diet, TRF does not produce the same microbial profile as CTRL-AL.

### 4.2. Diet and TRF Altered the Content and Composition of Fecal Fatty Acids

Fecal fatty acid content was dramatically affected by both dietary fatty acid content and by feeding timing. Total fecal fatty acids were doubled by HF-AL compared to CTRL-AL, but HF-TRF more than doubled the fecal fatty acid content of HF-AL. Previously published data have also demonstrated elevated fecal fatty acid content from HF diets compared to LF diets, but to our knowledge, the effect of HF-TRF on fecal fatty acid content has not been reported [42,43]. This finding may explain in part why TRF diets lower body weight and adiposity and improve metabolic function. The relative abundance of dietary fatty acids differed from that of fecal fatty acids. SFA were the most abundant fecal fatty acids for each dietary intervention and mirrored total fecal fatty acid content. However, the percentage of fecal SFA across dietary interventions was nearly 1.7-fold higher than the percent SFA content of each diet. These findings are consistent with previously reported data showing reduced absorption of SFA compared to unsaturated fats [44,45,46]. The energy intake of the HF-TRF animals did not differ from the HF-AL group pointing to a saturable absorption mechanism in which unsaturated fats are more effectively absorbed than saturated fats.

### 4.3. Diet and TRF Affected Bile Acids and SCFAs Associated with Microbial Metabolism

First, although total bile acid content did not differ between diets, the most abundant fecal bile acid, DCA, was nonstatistically decreased (*p* = 0.08) in HF-TRF compared to HF-AL. DCA and other 12-α hydroxylated bile acids are associated with insulin resistance [47]. Thus, the lack of differences in total fecal bile acids may indicate insufficient bile acids production to digest and absorb dietary fat, while decreased DCA may be related to the improvements in glucose homeostasis observed in TRF. On the other hand, SCFA content was altered by both fat content and feeding timing. Total SCFAs were elevated in CTRL-AL compared to HF-AL but not HF-TRF, likely due to the ability of the colonic ecosystem of the TRF animals to rely on mucosal glycoproteins for energy [48]. Acetate and propionate production was promoted by CTRL-AL feeding, whereas TRF promoted butyrate production, which may explain the improved insulin sensitivity by TRF [25] because butyrate is linked to improvements in glucose homeostasis [49].

Second, SCFAs were related to bacterial taxonomic classifications, particularly in the CTRL-AL diet. Bacteroidetes favored fecal SCFA concentrations, while Firmicutes inhibited SCFAs in the CTRL-AL diet. The percent acetate decreased in CTRL-AL, but the relative abundance of butyrate increased with Bacteroidetes. Interestingly at the family level in CTRL-AL, *Desulfovibrionaceae* increased with percent acetate but decreased with percent butyrate. *Desulfovibrionaceae* was inversely related to the concentrations of SCFAs. The CTRL-AL diet had higher starch content than the HF diets and likely provided undigested substrate to the microbiota of the CTRL-AL animals, which are converted to SCFAs. The inverse relationship between SCFAs and *Desulfovibrionaceae* points to ecological crosstalk within the environment of the gut in which presumptively healthy metabolites like SCFAs either inhibit the viability of taxa like those within *Desulfovibrionaceae* or are reflective of the proliferation of commensal taxa at the expense of pathogenic taxa.

Lastly, HF diets did demonstrate effects on the fecal microbiota at the family level. HF-TRF promoted the relationships between two families in Firmicutes, *Clostridiaceae* 1 and *Peptococcaceae,* with SCFAs where the percent acetate decreased, and the percent butyrate increased with each. In HF-AL, we observed inverse relationships between SCFAs and the families *Eggerthellaceae* and *Flavobacteriaceae* in Actinobacteria and Bacteroidetes, respectively. These data highlight the importance of looking deeper into the phylogenic tree to discern the effects of diet on gut microbial ecology and its metabolites than the phyla.

There are limitations to our data in this report. First, the use of a purified diet in our animal model is different from the dietary heterogeneity of people. Second, more work is needed to examine the relationship between fecal bile acid content and absorbed bile acids. The fecal collections were only conducted in the dark phase of the day (active phase). A more detailed collection of feces (e.g., for background levels of fecal lipid, bile acid, and microbiome) would give a clearer picture of the relationship between fecal lipids, the fecal microbiota, and ingestion.

## 5. Conclusions

Time restriction in a high-fat diet alters the fecal microbial community in mice and profoundly changes the lipid metabolome, including increasing the fatty acid content of the feces. Although fecal concentrations of fatty acids were elevated in TRF, this did not correspond to elevations in fecal bile acids in this mouse model. It is possible that bile acid secretion and production are insufficient to aid in the digestion and absorption of dietary fat when consumed in a limited time frame. Moreover, HF diets and shortened feeding times in TRF resulting in poor absorption of dietary fat, particularly SFA, also could explain the differences in fecal fatty acids as well as the amelioration of obesity we observed in our previous analysis. Tracer studies are needed to determine the degree to which TRF may limit fatty acid absorption vs. enhancing the microbial synthesis of lipids. Given the frequent use of high-fat obesogenic diets in animal models for obesity research, it is imperative that we understand the impact of TRF on lipid metabolism in the gut.

## Data Availability

The data presented in this study are available on request from the corresponding author.

## Supplementary Materials

The following supporting information can be downloaded at: https://www.mdpi.com/article/10.3390/nu15071562/s1, Table S1: Spearman’s rho values between fecal microbial phyla and fatty acids for each diet: CTRL-AL, HF-AL, and HF-TRF; Table S2: Spearman’s rho values between fecal microbial families and fatty acids; Table S3: Spearman’s rho values for fecal microbial phyla by bile acids; Table S4: Spearman’s rho for fecal microbial families by bile acids; Table S5: Spearman’s rho values for fecal microbial phyla by short-chain fatty acids; Table S6: Spearman’s rho for fecal microbial families by short-chain fatty acids.
