# Supplementary material for: Time-Restricted Feeding Modifies the Fecal Lipidome and the Gut Microbiota
Source: Nutrients. 2023 Mar 23;15(7):1562. doi: 10.3390/nu15071562 (PMC10096715; doi:10.3390/nu15071562)
Supplement: Supplementary file 1 [file nutrients-15-01562-s001.zip › nutrients-2286714-supplementary.pdf]

Supplemental Table S1. Spearman's rho values between fecal microbial phyla and fatty acids for each diet: CTRL-AL, HF-AL, and HF-TRF (n=14 for each group). Blue shading highlights statistically significant negative Spearman's Rho. Green shading indicates positive associations. Normal font indicates $p < 0.05$; bold font indicates $p < 0.01$.

	Actinobacteria			Bacteroidetes			Deferribacteres			Firmicutes			Verrucomicrobia		
**SFA**	CTRL-AL	HF-AL	HF-TRF	CTRL-AL	HF-AL	HF-TRF	CTRL-AL	HF-AL	HF-TRF	CTRL-AL	HF-AL	HF-TRF	CTRL-AL	HF-AL	HF-TRF
12:0	-0.34	-0.60	-0.01	0.30	0.63	0.13	-0.11	-0.02	-0.43	-0.03	-0.30	-0.15	-0.27	-0.24	0.27
14:0	-0.05	-0.54	0.07	0.44	0.61	0.10	-0.48	0.04	-0.42	-0.43	-0.27	-0.16	-0.18	-0.11	0.38
15:0	0.11	-0.29	-0.09	0.31	0.34	0.43	-0.52	0.20	**-0.67**	-0.35	-0.21	-0.53	-0.22	0.25	0.39
16:0	-0.09	-0.57	0.03	0.54	0.58	0.08	-0.57	-0.09	-0.42	-0.44	-0.28	-0.13	-0.37	-0.13	0.34
17:0	-0.16	-0.59	-0.01	0.46	0.62	0.09	-0.54	-0.05	-0.48	-0.36	-0.32	-0.18	-0.18	-0.12	0.41
18:0	-0.10	-0.61	0.00	0.55	0.60	0.11	-0.59	-0.09	-0.46	-0.50	-0.32	-0.13	-0.29	-0.14	0.31
20:0	-0.25	-0.53	-0.03	0.60	0.58	0.01	-0.57	-0.17	-0.44	**-0.69**	-0.37	-0.06	0.11	-0.11	0.31
21:0	0.07	-0.11	-0.14	-0.07	-0.04	-0.15	0.02	-0.10	-0.55	-0.10	-0.15	0.07	0.42	0.06	0.17
23:0	-0.39	-0.41	-0.16	0.40	0.17	-0.08	-0.34	0.03	-0.53	-0.35	-0.05	0.07	-0.01	-0.08	0.13
24:0	-0.26	-0.47	-0.10	0.48	0.33	-0.09	-0.40	-0.04	-0.49	-0.52	-0.17	0.06	0.19	-0.13	0.22
**Total SFA**	-0.10	-0.57	0.02	0.58	0.59	0.08	-0.58	-0.09	-0.43	-0.49	-0.30	-0.12	-0.29	-0.11	0.32
**BCFA**															
13:0, 12-Me	-0.20	-0.09	-0.17	0.47	0.42	0.17	-0.51	0.00	-0.30	-0.56	-0.61	-0.63	0.56	**0.72**	**0.88**
14:0, 13-Me	-0.14	-0.38	-0.03	**0.69**	0.36	0.18	-0.60	0.17	-0.39	**-0.69**	-0.30	-0.52	-0.03	0.59	**0.79**
14:0, 12-Me	-0.01	-0.27	-0.11	0.49	0.59	0.21	**-0.67**	-0.10	-0.37	-0.60	-0.60	-0.58	0.05	0.63	**0.81**
15:0, 14-Me	0.02	-0.64	0.10	0.35	0.56	0.08	-0.34	0.17	-0.55	-0.35	-0.45	-0.34	-0.02	0.27	**0.69**
16:0, 15-Me	0.06	-0.37	0.07	0.33	0.29	0.37	-0.40	0.12	**-0.80**	-0.13	-0.28	-0.44	-0.53	0.51	0.36
16:0, 14-Me	-0.08	-0.48	0.20	0.44	**0.79**	0.47	-0.28	-0.18	-0.39	-0.36	-0.60	-0.58	-0.10	0.17	0.44
17:0, 16-Me	-0.02	-0.34	0.08	0.59	**0.66**	0.24	-0.52	-0.12	**-0.67**	-0.58	-0.59	-0.23	-0.18	0.47	0.16
21:0, 18-Me	-0.11	-0.65	0.13	0.58	0.57	-0.12	-0.58	0.06	-0.27	**-0.68**	-0.30	-0.01	0.26	-0.17	0.45
**Total BCFA**	-0.05	-0.42	0.13	0.55	0.64	0.26	-0.64	-0.04	-0.45	-0.62	-0.62	-0.49	0.08	0.61	**0.69**
**MUFA**															
16:1n-7, Z	0.09	-0.34	0.11	0.52	**0.73**	0.49	-0.58	-0.15	-0.64	-0.51	-0.63	-0.59	-0.12	0.34	0.43
18:1n-9,E	-0.26	-0.56	-0.27	0.65	0.65	0.08	-0.64	0.07	**-0.74**	-0.57	-0.43	-0.07	0.03	0.23	0.19
18:1n-9,Z	-0.13	-0.59	-0.01	0.42	0.65	0.16	-0.42	-0.03	-0.47	-0.30	-0.35	-0.15	-0.19	-0.12	0.26
18:1n-7, Z	0.12	-0.49	0.01	0.32	**0.75**	0.31	-0.50	-0.09	-0.62	-0.32	-0.56	-0.38	-0.33	0.23	0.41
18:1n-6, Z	0.39	-0.01	-0.07	-0.17	0.41	0.29	-0.22	0.02	-0.18	0.05	-0.43	-0.50	-0.22	0.37	0.51
20:1n-9	-0.08	-0.65	0.07	0.56	0.54	-0.06	-0.35	0.05	-0.34	**-0.75**	-0.32	-0.06	0.23	-0.10	0.43
22:1n-9	-0.50	0.06	0.25	0.41	-0.21	0.17	-0.27	0.31	-0.43	-0.25	0.21	-0.07	0.46	-0.08	-0.10
24:1n-9	-0.10	-0.41	-0.22	0.35	-0.08	-0.16	-0.39	0.35	-0.48	-0.46	0.11	0.13	0.14	-0.06	0.06
**Total MUFA**	-0.22	-0.63	-0.10	0.53	0.64	0.19	-0.60	0.04	-0.55	-0.46	-0.28	-0.21	-0.12	-0.10	0.32
**n-6 PUFA**															
18:2, 9Z,11E	0.12	-0.17	0.12	0.29	0.15	-0.30	-0.29	-0.21	-0.39	-0.45	-0.26	0.24	0.19	-0.06	0.02
18:2 Z/Z	0.16	0.06	-0.01	-0.42	0.18	0.08	0.04	0.14	-0.32	0.31	-0.32	-0.22	-0.29	0.10	0.22
18:2n-6, Z,Z	-0.02	-0.44	0.12	0.19	0.55	0.12	-0.25	-0.18	-0.32	-0.18	-0.26	-0.19	-0.23	-0.16	0.39
20:3n-6	-0.39	-0.49	-0.14	0.49	0.40	-0.21	-0.53	0.03	-0.42	-0.47	-0.28	0.16	0.19	-0.04	0.22
20:4n-6	0.17	-0.38	0.04	0.16	0.33	-0.45	-0.10	-0.05	-0.12	-0.42	-0.28	0.19	0.47	-0.04	0.46
22:4n-6	-0.11	-0.45	0.41	0.23	0.09	-0.02	-0.33	0.15	-0.04	-0.40	-0.05	0.15	0.27	0.21	-0.08
22:5n-6	0.03	0.04	-0.19	0.10	0.00	-0.04	-0.52	-0.36	-0.56	-0.31	-0.11	0.05	0.04	-0.05	0.12
**Total n-6 PUFA**	-0.11	-0.56	0.07	0.37	0.57	-0.05	-0.53	-0.08	-0.32	-0.45	-0.37	-0.04	0.17	-0.07	0.41
**n-3 PUFA**															
18:3n-3	-0.11	-0.49	0.04	0.23	0.55	0.13	-0.22	-0.18	-0.40	-0.21	-0.20	-0.16	-0.12	-0.29	0.31
20:5n-3	0.02	-0.10	0.14	0.16	0.00	-0.34	-0.37	0.06	-0.31	-0.25	-0.15	0.30	0.12	0.08	-0.05
22:5n-3	-0.17	0.16	0.26	0.18	-0.11	0.18	-0.35	-0.05	-0.31	-0.39	0.23	-0.27	0.24	0.22	0.51
22:6n-3	-0.31	0.31	0.11	0.22	-0.28	-0.18	-0.35	0.04	-0.47	-0.24	0.12	0.24	0.45	-0.01	-0.19

**Total n-3 PUFA**	-0.09	-0.49	0.13	0.18	0.52	-0.11	-0.39	-0.15	-0.29	-0.25	-0.25	0.03	0.23	-0.24	0.37
**Total FA**	-0.14	-0.58	0.02	0.64	0.59	0.08	-0.63	-0.07	-0.43	-0.59	-0.28	-0.12	-0.14	-0.12	0.32

AL, ad libitum; CTRL, control diet; HF, high fat diet; TRF, time-restricted feeding; BCFA, branched-chain fatty acids; FA, fatty acids;MUFA, monounsaturated fatty acids; PUFA, polyunsaturated fatty acids; SFA, saturated fatty acids.

Supplemental Table S2. Spearman's rho values between fecal microbial families and fatty acids. Blue shading highlights statistically significant negative Spearman's Rho. Green shading indicates positive associations. Normal font indicates p < 0.05; bold font indicates p < 0.01.

	Bifidobacteriaceae			*Bacteroidaceae*			*Tannerellaceae*			*Deferribacteraceae*			*Clostridiaceae*			*Lachnospiraceae*			*Desulfovibrionaceae*			*Akkermansiaceae*		
**SFA**	CTRL-AL	HF-AL	HF-TRF	CTRL-AL	HF-AL	HF-TRF	CTRL-AL	HF-AL	HF-TRF	CTRL-AL	HF-AL	HF-TRF	CTRL-AL	HF-AL	HF-TRF	CTRL-AL	HF-AL	HF-TRF	CTRL-AL	HF-AL	HF-TRF	CTRL-AL	HF-AL	HF-TRF
12:0	-0.22	-0.65	NA	0.30	0.64	0.29	0.06	0.28	0.02	-0.11	-0.02	-0.43	0.33	0.32	**0.83**	0.22	-0.06	0.01	-0.26	-0.56	-0.40	-0.27	-0.24	0.27
14:0	0.02	-0.55	NA	0.57	**0.67**	0.29	0.36	0.31	0.00	-0.48	0.04	-0.42	0.50	0.35	**0.83**	-0.21	-0.05	0.01	-0.52	-0.57	-0.41	-0.18	-0.11	0.38
15:0	0.10	-0.30	NA	0.58	0.50	0.43	0.48	**0.71**	0.44	-0.52	0.20	**-0.67**	0.27	-0.06	0.03	-0.45	-0.46	-0.39	-0.34	-0.03	**-0.71**	-0.22	0.25	0.39
16:0	0.02	-0.62	NA	0.60	0.63	0.27	0.44	0.25	-0.01	-0.57	-0.09	-0.42	0.51	0.30	**0.82**	-0.22	0.02	0.04	-0.55	-0.56	-0.42	-0.37	-0.13	0.34
17:0	-0.21	-0.65	NA	0.59	**0.71**	0.31	0.33	0.41	0.01	-0.54	-0.05	-0.48	0.49	0.28	**0.78**	-0.35	-0.05	0.00	-0.49	-0.53	-0.48	-0.18	-0.12	0.41
18:0	-0.01	-0.64	NA	0.56	0.65	0.28	0.38	0.28	0.01	-0.59	-0.09	-0.46	0.56	0.33	**0.82**	-0.35	-0.01	0.03	-0.57	-0.56	-0.44	-0.29	-0.14	0.31
20:0	-0.18	-0.59	NA	0.61	0.65	0.24	0.56	0.34	-0.05	-0.57	-0.17	-0.44	0.27	0.26	**0.79**	**-0.73**	-0.05	0.11	-0.57	-0.51	-0.43	0.11	-0.11	0.31
21:0	-0.08	-0.05	NA	-0.01	0.17	0.04	0.08	0.20	0.00	0.02	-0.10	-0.55	-0.42	-0.01	0.31	-0.54	0.14	0.22	0.14	0.11	-0.63	0.42	0.06	0.17
23:0	-0.31	-0.47	NA	0.48	0.40	0.07	0.42	0.34	-0.03	-0.34	0.03	-0.53	0.26	-0.04	0.44	**-0.66**	0.16	0.23	-0.36	0.01	-0.63	-0.01	-0.08	0.13
24:0	-0.20	-0.57	NA	0.51	0.48	0.12	0.54	0.34	-0.09	-0.40	-0.04	-0.49	0.09	0.00	0.58	**-0.73**	0.09	0.24	-0.44	-0.13	-0.59	0.19	-0.13	0.22
Total	0.01	-0.62	NA	0.66	0.64	0.26	0.51	0.27	-0.01	-0.58	-0.09	-0.43	0.46	0.32	**0.81**	-0.31	0.00	0.05	-0.58	-0.58	-0.43	-0.29	-0.11	0.32
**BCFA**																								
13:0, 12-Me	-0.29	0.22	NA	**0.68**	0.41	0.34	0.48	0.50	0.44	-0.51	0.00	-0.30	0.05	0.37	-0.03	**-0.70**	-0.61	-0.53	-0.46	-0.26	-0.39	0.56	**0.72**	**0.88**
14:0, 13-Me	-0.08	-0.12	NA	**0.82**	0.50	0.27	**0.73**	0.46	0.36	-0.60	0.17	-0.39	0.28	0.07	0.22	-0.56	-0.29	-0.37	**-0.69**	-0.17	-0.46	-0.03	0.59	**0.79**
14:0, 12-Me	0.09	-0.06	NA	**0.78**	**0.73**	0.35	**0.73**	**0.69**	0.43	**-0.67**	-0.10	-0.37	0.13	0.45	0.09	-0.50	-0.57	-0.43	-0.49	-0.42	-0.54	0.05	0.63	**0.81**
15:0, 14-Me	0.00	-0.46	NA	0.40	**0.67**	0.24	0.33	**0.68**	0.15	-0.34	0.17	-0.55	0.26	0.40	0.37	-0.58	-0.37	-0.18	-0.39	-0.30	-0.51	-0.02	0.27	**0.69**
16:0, 15-Me	0.12	-0.13	NA	0.28	0.38	0.37	0.34	0.46	0.45	-0.40	0.12	**-0.80**	0.43	0.02	0.37	-0.07	-0.09	-0.36	-0.40	-0.14	**-0.67**	-0.53	0.51	0.36
16:0, 14-Me	-0.04	-0.48	NA	0.46	**0.77**	0.61	0.36	**0.73**	0.64	-0.28	-0.18	-0.39	0.28	0.09	0.15	-0.30	-0.43	-0.59	-0.53	-0.51	-0.58	-0.10	0.17	0.44
17:0, 16-Me	-0.05	-0.30	NA	0.64	0.59	0.28	0.55	0.65	0.23	-0.52	-0.12	**-0.67**	0.33	0.29	0.33	-0.47	-0.51	-0.14	-0.57	-0.42	-0.64	-0.18	0.47	0.16
21:0, 18-Me	0.03	-0.63	NA	0.66	0.63	0.19	0.61	0.39	-0.13	-0.58	0.06	-0.27	0.24	0.25	**0.78**	-0.47	0.00	0.15	-0.62	-0.46	-0.35	0.26	-0.17	0.45
Total	-0.04	-0.20	NA	**0.80**	**0.72**	0.26	0.64	**0.67**	0.30	-0.64	-0.04	-0.45	0.23	0.29	0.24	-0.54	-0.53	-0.35	-0.57	-0.39	-0.44	0.08	0.61	**0.69**
**MUFA**																								
16:1n-7, Z	0.05	-0.36	NA	0.60	**0.67**	0.51	0.52	**0.71**	0.45	-0.58	-0.15	-0.64	0.32	0.24	0.20	-0.41	**-0.68**	-0.44	-0.54	-0.42	**-0.71**	-0.12	0.34	0.43
18:1n-9,E	-0.23	-0.44	NA	**0.78**	**0.79**	0.09	0.63	**0.76**	0.07	-0.64	0.07	**-0.74**	0.29	0.32	0.39	-0.48	-0.31	0.10	-0.60	-0.38	-0.62	0.03	0.23	0.19
18:1n-9,Z	-0.11	-0.63	NA	0.58	**0.69**	0.29	0.31	0.40	0.02	-0.42	-0.03	-0.47	0.31	0.26	**0.85**	-0.07	-0.12	0.00	-0.33	-0.53	-0.41	-0.19	-0.12	0.26
18:1n-7, Z	0.06	-0.35	NA	0.31	**0.87**	0.46	0.17	0.52	0.21	-0.50	-0.09	-0.62	0.54	0.14	**0.71**	-0.13	-0.37	-0.22	-0.39	-0.59	-0.58	-0.33	0.23	0.41
18:1n-6, Z	0.35	-0.04	NA	-0.16	0.29	0.45	-0.09	0.56	0.23	-0.22	0.02	-0.18	0.36	0.41	0.09	-0.06	-0.61	-0.35	-0.07	-0.33	-0.35	-0.22	0.37	0.51
20:1n-9	-0.02	-0.58	NA	**0.70**	**0.66**	0.22	0.60	0.43	-0.07	-0.35	0.05	-0.34	0.02	0.26	**0.75**	-0.55	0.01	0.10	-0.48	-0.38	-0.41	0.23	-0.10	0.43
22:1n-9	-0.52	0.10	NA	0.43	0.08	-0.04	0.31	0.27	0.37	-0.27	0.31	-0.43	-0.10	-0.12	-0.34	-0.33	-0.02	-0.17	-0.20	0.49	-0.46	0.46	-0.08	-0.10
24:1n-9	-0.17	-0.35	NA	0.53	0.14	-0.11	0.49	0.37	-0.04	-0.39	0.35	-0.48	0.00	-0.16	0.24	**-0.80**	0.12	0.28	-0.22	0.37	-0.57	0.14	-0.06	0.06
Total	-0.18	-0.65	NA	**0.73**	**0.69**	0.31	0.46	0.40	0.08	-0.60	0.04	-0.55	0.41	0.23	**0.79**	-0.32	-0.08	-0.06	-0.46	-0.54	-0.45	-0.12	-0.10	0.32
**n-6 PUFA**																								
18:2, 9Z,11E	0.18	-0.24	NA	0.31	0.18	-0.06	0.52	0.24	-0.13	-0.29	-0.21	-0.39	-0.12	0.09	0.33	-0.59	0.06	0.35	-0.28	-0.08	-0.45	0.19	-0.06	0.02
18:2 Z/Z	0.35	0.36	NA	-0.40	0.22	0.06	-0.17	0.34	0.17	0.04	0.14	-0.32	0.22	0.19	-0.23	0.09	-0.43	-0.19	0.22	-0.10	-0.14	-0.29	0.10	0.22
18:2n-6, Z,Z	0.06	-0.60	NA	0.37	0.52	0.35	0.09	0.24	0.02	-0.25	-0.18	-0.32	0.21	0.27	**0.90**	0.29	-0.04	-0.05	-0.07	-0.54	-0.32	-0.23	-0.16	0.39
20:3n-6	-0.35	-0.55	NA	0.49	0.57	-0.04	0.44	0.58	-0.14	-0.53	0.03	-0.42	0.28	0.19	0.48	**-0.71**	-0.16	0.34	-0.46	-0.12	-0.53	0.19	-0.04	0.22
20:4n-6	0.15	-0.35	NA	0.25	0.44	-0.09	0.34	0.37	-0.27	-0.10	-0.05	-0.12	-0.30	0.04	0.46	-0.52	0.02	0.36	-0.09	-0.29	-0.23	0.47	-0.04	0.46
22:4n-6	-0.21	-0.40	NA	0.25	0.35	-0.03	0.14	0.52	-0.08	-0.33	0.15	-0.04	0.19	0.16	0.17	**-0.73**	0.05	0.14	-0.20	0.11	-0.18	0.27	0.21	-0.08
22:5n-6	0.02	-0.16	NA	0.20	0.01	0.07	0.14	-0.05	-0.02	-0.52	-0.36	-0.56	0.33	0.01	0.48	-0.61	0.28	0.21	-0.19	-0.07	-0.62	0.04	-0.05	0.12
Total	-0.10	-0.59	NA	0.65	**0.68**	0.23	0.36	0.42	-0.10	-0.53	-0.08	-0.32	0.15	0.08	**0.80**	-0.34	-0.10	0.13	-0.29	-0.42	-0.38	0.17	-0.07	0.41
**n-3 PUFA**																								
18:3n-3	-0.01	**-0.70**	NA	0.37	0.50	0.34	0.10	0.16	0.01	-0.22	-0.18	-0.40	0.15	0.31	**0.89**	0.28	0.05	-0.01	-0.13	-0.57	-0.37	-0.12	-0.29	0.31
20:5n-3	0.08	-0.12	NA	0.24	0.10	-0.16	0.25	0.45	-0.02	-0.37	0.06	-0.31	-0.01	0.02	0.12	-0.32	-0.15	0.28	-0.06	0.26	-0.32	0.12	0.08	-0.05
22:5n-3	-0.06	-0.02	NA	0.38	-0.16	0.24	0.13	0.01	0.12	-0.35	-0.05	-0.31	0.37	-0.14	0.30	-0.39	0.08	-0.10	-0.28	0.00	-0.33	0.24	0.22	0.51
22:6n-3	-0.24	0.30	NA	0.33	-0.12	-0.32	0.11	0.09	0.06	-0.35	0.04	-0.47	0.09	-0.22	-0.43	-0.13	0.03	0.15	-0.13	0.52	-0.39	0.45	-0.01	-0.19
Total	0.01	-0.53	NA	0.46	0.62	0.14	0.18	0.21	-0.12	-0.39	-0.15	-0.29	0.09	0.06	**0.73**	-0.04	0.08	0.19	-0.14	-0.42	-0.40	0.23	-0.24	0.37
**Total FA**	-0.04	-0.64	NA	**0.76**	0.63	0.26	0.60	0.27	-0.01	-0.63	-0.07	-0.43	0.40	0.32	**0.81**	-0.39	0.00	0.05	-0.63	-0.58	-0.43	-0.14	-0.12	0.32

AL, *ad libitum;* CTRL, control diet; HF, high fat diet; TRF, time-restricted feeding; FA, fatty acids; BCFA, branched chain fatty acids; MUFA, monounsaturated fatty acids; PUFA, polyunsaturated fatty acids; SFA, saturated fatty acids; NA, not applicable due to all zero relative abundance in HF-TRF.

Supplemental Table S3. Spearman's rho values for fecal microbial phyla by bile acids. Blue shading highlights statistically significant negative Spearman's Rho. Green shading indicates positive associations. Normal font indicates $p < 0.05$; bold font indicates $p < 0.01$.

	Actinobacteria			Bacteroidetes			Deferribacteres			Firmicutes			Patescibacteria			Verrucomicrobia		
Bile Acids	CTRL-AL	HF-AL	HF-TRF	CTRL-AL	HF-AL	HF-TRF	CTRL-AL	HF-AL	HF-TRF	CTRL-AL	HF-AL	HF-TRF	CTRL-AL	HF-AL	HF-TRF	CTRL-AL	HF-AL	HF-TRF
α-MCA	-0.20	0.30	0.39	0.24	-0.22	-0.34	-0.41	-0.30	**0.75**	-0.22	0.24	0.62	-0.59	-0.05	0.01	-0.24	0.00	-0.56
β/ω- MCA	-0.10	-0.18	0.50	0.24	0.15	-0.25	-0.10	-0.31	0.47	-0.09	0.12	0.47	-0.54	-0.15	-0.03	-0.14	-0.13	-0.38
DCA	0.02	0.43	-0.21	0.05	-0.17	-0.18	-0.55	-0.21	0.29	-0.05	0.10	0.45	0.17	0.34	0.47	-0.15	0.04	**-0.66**
t-DCA	-0.11	0.48	-0.28	0.25	-0.09	-0.53	-0.24	-0.43	0.39	-0.29	-0.25	0.58	-0.31	-0.02	0.11	0.07	0.45	-0.05
HDCA	0.00	0.29	-0.63	-0.15	0.27	0.12	-0.01	-0.12	-0.26	0.10	-0.47	-0.08	0.20	0.14	0.25	-0.14	0.26	-0.16
t-HDCA	-0.07	0.41	-0.62	-0.16	-0.17	-0.31	0.22	0.09	0.10	0.10	-0.19	0.39	0.08	0.16	0.35	-0.07	0.19	-0.43
t-CDCA	-0.18	0.40	-0.39	-0.02	-0.61	-0.47	0.04	0.31	0.37	-0.04	0.27	0.61	0.19	0.09	0.40	0.12	-0.01	-0.42
LCA	0.15	0.27	-0.43	-0.06	-0.20	-0.15	-0.51	0.06	-0.09	-0.03	0.08	0.44	0.24	-0.19	0.51	-0.34	0.17	-0.36
CA	-0.27	**0.68**	0.22	0.08	-0.02	-0.36	-0.23	**-0.72**	**0.68**	-0.04	-0.21	0.55	-0.21	-0.21	-0.02	0.02	0.45	-0.51
α-t-CA	-0.23	0.62	-0.27	0.09	-0.59	-0.52	-0.02	0.05	0.27	-0.08	0.25	**0.69**	0.06	0.11	0.30	-0.02	0.10	-0.51
t-CA	-0.22	0.61	-0.37	0.01	**-0.72**	-0.40	0.18	0.09	0.31	-0.04	0.42	0.52	0.08	0.15	0.43	0.14	-0.02	-0.42
ω-t-CA	0.25	0.06	-0.11	-0.29	-0.48	-0.52	0.33	0.28	0.08	0.19	0.35	**0.73**	0.01	0.09	0.11	0.12	-0.02	-0.40
UDCA	0.10	0.38	-0.41	0.03	**-0.66**	0.30	-0.52	-0.05	0.07	-0.01	0.50	-0.09	0.10	0.14	0.58	-0.18	0.16	-0.39
**Taurine conjugates**	-0.07	0.46	-0.30	-0.10	**-0.71**	-0.54	0.21	0.20	0.18	0.06	0.38	**0.69**	0.03	0.10	0.26	0.08	0.07	-0.41
**Total bile acids**	-0.13	0.21	0.00	0.22	-0.07	-0.24	-0.41	-0.36	0.49	-0.16	0.15	0.60	-0.27	0.17	0.41	-0.17	0.04	**-0.74**

AL, *ad libitum;* CTRL, control diet; HF, high fat diet; TRF, time-restricted feeding; α-MCA, α-muricholic acid; β/ω muricholic acids; DCA, deoxycholic acid; t-DCA, taurine deoxycholic acid; HDCA, hyodeoxycholic acid; t-HDCA, taurine hyodeoxycholic acid; t-CDCA, taurine chenodeoxycholic acid; LCA, lithocholic acid; CA, cholic acid; α-t-cholic acid, alpha taurine cholic acid; t-CA, taurine cholic acid; ω-t-cholic acid, alpha taurine cholic acid; UDCA, ursodeoxycholic acid.

Supplemental Table S4. Spearman's rho for fecal microbial families by bile acids. Blue shading highlights statistically significant negative Spearman's Rho. Green shading indicates positive associations. Normal font indicates $p < 0.05$; bold font indicates $p < 0.01$.

													Clostridiales											
	Bifidobacteriaceae			*Bacteroidaceae*			*Tannerellaceae*			*Deferribacteraceae*			*Family_XIII*			*Lachnospiraceae*			*Peptococcaceae*			*Akkermansiaceae*		
Bile Acids	CTRL-AL	HF-AL	HF-TRF	CTRL-AL	HF-AL	HF-TRF	CTRL-AL	HF-AL	HF-TRF	CTRL-AL	HF-AL	HF-TRF	CTRL-AL	HF-AL	HF-TRF	CTRL-AL	HF-AL	HF-TRF	CTRL-AL	HF-AL	HF-TRF	CTRL-AL	HF-AL	HF-TRF
α-MCA	-0.18	0.11	NA	0.24	-0.04	-0.33	0.14	-0.19	-0.51	-0.41	-0.30	**0.75**	-0.11	-0.03	0.29	-0.06	0.41	0.53	-0.12	-0.34	0.39	-0.24	0.00	-0.56
β/ω- MCA	-0.12	-0.43	NA	0.12	0.20	-0.24	0.12	-0.10	-0.34	-0.10	-0.31	0.47	-0.11	0.09	0.02	0.13	0.43	0.32	0.12	-0.20	0.47	-0.14	-0.13	-0.38
DCA	0.08	0.11	NA	0.09	-0.05	-0.20	0.16	0.28	-0.07	-0.55	-0.21	0.29	0.00	0.23	**0.69**	-0.34	-0.20	0.29	0.03	-0.66	-0.27	-0.15	0.04	**-0.66**
t-DCA	0.03	0.45	NA	0.31	-0.12	-0.37	0.34	0.02	-0.48	-0.24	-0.43	0.39	-0.01	0.33	0.28	-0.23	-0.06	0.57	0.33	-0.40	0.32	0.07	0.45	-0.05
HDCA	0.26	0.37	NA	-0.03	0.34	0.25	0.19	0.46	0.22	-0.01	-0.12	-0.26	-0.29	-0.01	0.38	0.03	**-0.78**	-0.02	0.00	-0.30	**-0.69**	-0.14	0.26	-0.16
t-HDCA	0.17	0.61	NA	-0.13	-0.16	-0.21	0.11	0.09	-0.10	0.22	0.09	0.10	-0.15	-0.05	**0.69**	0.09	-0.45	0.36	0.14	-0.07	-0.42	-0.07	0.19	-0.43
t-CDCA	-0.08	0.64	NA	0.05	-0.52	-0.55	0.22	-0.25	**-0.66**	0.04	0.31	0.37	-0.07	0.01	0.61	-0.32	0.09	0.66	0.18	-0.02	0.06	0.12	-0.01	-0.42
LCA	0.17	0.47	NA	0.03	0.03	-0.23	0.10	0.06	-0.35	-0.51	0.06	-0.09	-0.13	-0.13	0.29	-0.34	-0.10	0.49	-0.19	-0.14	-0.34	-0.34	0.17	-0.36
CA	-0.22	0.43	NA	-0.05	-0.08	-0.12	-0.04	-0.09	-0.50	-0.23	**-0.72**	**0.68**	0.23	0.51	0.22	-0.24	-0.02	0.48	0.10	-0.35	0.22	0.02	0.45	-0.51
α-t-CA	-0.18	**0.67**	NA	-0.04	-0.60	-0.63	0.10	-0.37	-0.61	-0.02	0.05	0.27	0.21	0.15	0.52	-0.35	0.04	0.66	0.22	-0.10	0.06	-0.02	0.10	-0.51
t-CA	-0.15	0.61	NA	-0.14	**-0.74**	-0.56	0.02	-0.55	-0.41	0.18	0.09	0.31	0.25	0.16	0.62	-0.23	0.19	0.48	0.49	-0.16	0.05	0.14	-0.02	-0.42
ω-t-CA	0.21	0.24	NA	-0.27	-0.22	-0.59	-0.14	-0.24	**-0.70**	0.33	0.28	0.08	0.02	-0.07	0.35	-0.07	0.31	**0.75**	**0.70**	0.10	0.21	0.12	-0.02	-0.40
UDCA	0.21	0.35	NA	0.07	-0.66	0.06	0.19	**-0.70**	0.44	-0.52	-0.05	0.07	-0.01	-0.02	0.45	-0.20	0.50	-0.28	-0.04	-0.34	-0.13	-0.18	0.16	-0.39
Taurine conjugated	-0.04	0.64	NA	-0.13	-0.59	-0.66	-0.02	-0.47	-0.63	0.21	0.20	0.18	0.04	0.04	0.51	-0.21	0.23	**0.69**	0.57	-0.04	0.13	0.08	0.07	-0.41
Total fecal BA	0.00	-0.08	NA	0.20	0.14	-0.19	0.32	0.02	-0.24	-0.41	-0.36	0.49	-0.10	0.08	0.55	-0.17	0.18	0.44	0.18	-0.66	-0.08	-0.17	0.04	**-0.74**

AL, *ad libitum;* CTRL, control diet; HF, high fat diet; TRF, time-restricted feeding; α-MCA, α-muricholic acid; β/ω muricholic acids; DCA, deoxycholic acid; t-DCA, taurine deoxycholic acid; HDCA, hyodeoxycholic acid; t-HDCA, taurine hyodeoxycholic acid; t-CDCA, taurine chenodeoxycholic acid; LCA, lithocholic acid; CA, cholic acid; α-t-cholic acid, alpha taurine cholic acid; t-CA, taurine cholic acid; ω-t-cholic acid, alpha taurine cholic acid; UDCA, ursodeoxycholic acid; NA, not applicable due to all zero relative abundance in HF-TRF.

Supplemental Table S5. Spearman's rho values for fecal microbial phyla by short chain fatty acids. SCFA, short chain fatty acids. Blue shading highlights statistically significant negative Spearman's Rho. Green shading indicates positive associations. Normal font indicates $p < 0.05$; bold font indicates $p < 0.01$.

Phylum	Actinobacteria			Bacteroidetes			Cyanobacteria			Deferribacteres			Firmicutes			Verrucomicrobia		
**SCFA**	CTRL-AL	HF-AL	HF-TRF	CTRL-AL	HF-AL	HF-TRF	CTRL-AL	HF-AL	HF-TRF	CTRL-AL	HF-AL	HF-TRF	CTRL-AL	HF-AL	HF-TRF	CTRL-AL	HF-AL	HF-TRF
% Acetate	0.53	0.54	-0.21	-0.54	-0.43	-0.14	-0.34	-0.05	-0.37	0.10	-0.05	-0.32	0.45	0.26	0.23	-0.34	-0.01	-0.42
% Propionate	-0.33	0.12	0.22	0.45	0.16	0.11	0.10	-0.14	0.60	0.09	-0.06	0.34	-0.42	-0.24	-0.39	0.38	0.22	0.62
% Butyrate	-0.51	-0.64	0.15	0.62	0.36	0.16	0.21	0.13	0.27	-0.30	0.02	0.27	-0.58	-0.18	-0.12	0.36	0.01	0.20
acetate	0.22	0.01	0.13	0.48	0.01	0.12	-0.13	0.07	0.18	-0.42	0.06	0.19	-0.58	-0.27	-0.07	0.01	0.31	0.20
propionate	-0.23	0.12	0.19	0.63	0.05	0.23	0.10	-0.07	0.46	-0.15	-0.07	0.31	-0.63	-0.25	-0.27	0.26	0.32	0.37
butyrate	-0.12	-0.41	0.19	0.64	0.28	0.22	0.13	0.17	0.25	-0.31	0.06	0.24	-0.65	-0.32	-0.16	0.19	0.13	0.19
isobutyrate	-0.09	0.16	0.14	**0.68**	-0.09	0.30	0.15	-0.08	0.07	-0.32	0.02	0.07	-0.53	-0.12	-0.15	0.12	0.42	-0.10
isovalerate	-0.24	0.25	0.11	0.63	-0.26	0.32	0.48	0.15	-0.11	-0.32	0.13	-0.16	-0.45	-0.03	-0.08	0.03	0.32	-0.20
valerate	0.04	0.05	0.17	0.59	0.02	0.22	-0.03	0.10	0.00	-0.41	-0.08	0.14	-0.63	-0.29	-0.02	0.11	0.49	-0.15
**Total SCFA**	0.06	-0.01	0.16	0.59	0.07	0.21	-0.09	0.09	0.21	-0.40	0.04	0.23	-0.66	-0.30	-0.15	0.08	0.29	0.19

AL, *ad libitum;* CTRL, control; HF, high fat diet; TRF, time-restricted feeding; SCFA, short chain fatty acids.

Supplemental Table S6. Spearman's rho for fecal microbial families by short chain fatty acids. Blue shading highlights statistically significant negative Spearman's Rho. Green shading indicates positive associations. Normal font indicates $p < 0.05$; bold font indicates $p < 0.01$.

Family	*Eggerthellaceae*			*Bacteroidaceae*			*Flavobacteriaceae*			*Muribaculaceae*			*Clostridiaceae_1*			*Clostridiales Family_XIII*			*Lactobacillaceae*			*Peptococcaceae*			*Desulfovibrionaceae*			*Enterobacteriaceae*			*Uncultured (Alpha) Proteobacteria*			*Tannerellaceae*		
**SCFA**	CTRL-	HF-AL	HF-TRF	CTRL-	HF-AL	HF-TRF	CTRL-	HF-AL	HF-TRF	CTRL-	HF-AL	HF-TRF	CTRL-	HF-AL	HF-TRF	CTRL-	HF-AL	HF-TRF	CTRL-	HF-AL	HF-TRF	CTRL-	HF-AL	HF-TRF	CTRL-	HF-AL	HF-TRF	CTRL-	HF-AL	HF-TRF	CTRL-	HF-AL	HF-TRF	CTRL-	HF-AL	HF-TRF
% Acetate	0.05	0.21	-0.18	-0.49	-0.51	-0.24	-0.37	0.52	0.27	-0.20	-0.15	-0.39	0.03	-0.06	-0.60	-0.10	0.39	0.34	-0.37	0.60	-0.10	-0.40	0.35	-0.55	0.58	0.33	-0.06	**0.38**	0.03	0.23	-0.43	-0.15	-0.23	-0.50	-0.11	0.03
% Propionate	0.13	-0.29	0.28	0.53	0.46	0.16	0.59	-0.26	-0.09	0.23	-0.15	0.32	-0.21	-0.18	0.11	-0.01	-0.35	-0.43	0.48	-0.16	0.31	0.35	-0.20	0.56	-0.46	0.08	0.01	-0.56	-0.32	-0.19	0.23	0.22	0.35	0.48	0.32	0.13
% Butyrate	0.05	-0.14	0.10	0.59	0.30	0.17	0.10	-0.45	-0.17	0.19	0.15	0.30	0.01	0.04	**0.71**	0.12	-0.25	-0.24	0.31	-0.55	0.04	0.24	-0.22	0.44	-0.58	-0.35	0.12	-0.16	0.14	0.00	0.52	0.03	0.13	0.59	-0.10	-0.08
acetate	-0.08	-0.61	0.12	0.52	0.26	0.01	-0.12	-0.56	-0.40	0.25	-0.20	0.53	0.11	-0.18	0.53	0.23	-0.58	-0.15	0.13	-0.32	0.18	-0.33	0.10	0.54	-0.47	0.16	-0.07	-0.14	-0.43	-0.15	0.58	0.14	0.25	0.57	-0.04	-0.06
propionate	-0.02	-0.44	0.23	**0.67**	0.35	0.19	0.42	-0.45	-0.27	0.34	-0.23	0.52	-0.09	-0.23	0.46	0.09	-0.47	-0.25	0.48	-0.23	0.28	0.03	-0.17	0.55	-0.62	0.12	-0.03	-0.46	-0.38	-0.18	0.51	0.16	0.39	**0.71**	0.10	0.03
butyrate	-0.09	-0.37	0.16	0.60	0.39	0.10	0.25	**-0.67**	-0.27	0.34	0.01	0.50	0.06	-0.08	0.63	0.30	-0.51	-0.20	0.38	-0.57	0.14	0.00	-0.11	0.51	**-0.68**	-0.16	0.05	-0.41	-0.10	0.00	**0.70**	0.09	0.25	**0.67**	-0.03	-0.02
isobutyrate	-0.15	-0.31	0.05	0.60	0.15	0.08	0.12	-0.06	-0.48	0.12	-0.58	0.49	0.00	-0.60	0.18	0.16	-0.38	0.13	0.35	-0.03	0.19	-0.19	0.18	0.03	**-0.69**	0.29	-0.33	-0.43	-0.12	-0.10	**0.72**	-0.17	0.31	**0.72**	0.04	0.06
isovalerate	-0.47	-0.50	0.05	0.38	-0.04	-0.07	0.15	-0.44	-0.43	0.17	-0.29	0.57	0.17	-0.38	0.11	0.30	-0.64	0.13	0.29	-0.15	-0.01	-0.10	0.11	-0.06	**-0.74**	0.36	-0.27	-0.39	-0.52	-0.04	**0.77**	0.20	0.22	0.61	-0.14	-0.01
valerate	-0.08	-0.53	0.09	0.61	0.26	-0.01	0.07	-0.63	-0.42	0.25	-0.30	0.52	0.10	-0.26	0.36	0.22	-0.57	0.05	0.28	-0.30	0.14	-0.15	-0.11	0.22	-0.61	0.05	-0.10	-0.25	-0.42	-0.02	**0.67**	0.16	0.16	0.66	-0.06	-0.08
**Total SCFA**	-0.05	-0.54	0.14	0.64	0.31	0.10	0.07	-0.59	-0.38	0.31	-0.19	0.55	0.11	-0.20	0.52	0.20	-0.58	-0.13	0.30	-0.33	0.20	-0.26	0.02	0.51	-0.59	0.10	-0.04	-0.25	-0.38	-0.15	0.62	0.13	0.30	**0.66**	-0.01	-0.01

SCFA, short chain fatty acids; AL, *ad libitum;* CTRL, control; HF, high fat diet; TRF, time-restricted feeding; SCFA, short chain fatty acids.
